# Dark-exciton valley dynamics in transition metal dichalcogenide alloy monolayers

**DOI:** 10.1038/s41598-019-40932-9

**Published:** 2019-03-14

**Authors:** Helena Bragança, Flávio Riche, Fanyao Qu, Victor Lopez-Richard, Gilmar Eugenio Marques

**Affiliations:** 1 Departamento de Física, Universidade Federal de São Carlos, São Carlos, SP, 13565-905 Brazil; 2 Instituto de Física, Universidade de Brasília, Brasília-DF, 70919-970 Brazil

## Abstract

We report a comprehensive theory to describe exciton and biexciton valley dynamics in monolayer Mo_1−*x*_W_*x*_Se_2_ alloys. To probe the impact of different excitonic channels, including bright and dark excitons, intravalley biexcitons, intervalley scattering between bright excitons, as well as bright biexcitons, we have performed a systematic study from the simplest system to the most complex one. In contrast to the binary WSe_2_ monolayer with weak photoluminescence (PL) and high valley polarization at low temperatures and the MoSe_2_, that presents high PL intensity, but low valley polarization, our results demonstrate that it is possible to set up a ternary alloy with intermediate W-concentration that holds simultaneously a considerably robust light emission and an efficient optical orientation of the valley pseudospin. We find the critical value of W-concentration, *x*_*c*_, that turns alloys from bright to darkish. The dependence of the PL intensity on temperature shows three regimes: while bright monolayer alloys display a usual temperature dependence in which the intensity decreases with rising temperature, the darkish alloys exhibit the opposite behavior, and the alloys with *x* around *x*_*c*_ show a non-monotonic temperature response. Remarkably, we observe that the biexciton enhances significantly the stability of the exciton emission against fluctuations of W-concentration for bright alloys. Our findings pave the way for developing high-performance valleytronic and photo-emitting devices.

## Introduction

Monolayers of transition metal dichalcogenides (TMDs), denoted by MX_2_ (M = Mo, W; X = S, Se, Te), have been attracting extreme attention from both experimental and theoretical point of views^1–4^. These promising materials are semiconductors with direct band gaps in the visible light energy range at two inequivalent K and K′ momentum valleys located at the edges of the hexagonal Brillouin zone^5,6^. The lattice inversion asymmetry combined with strong spin-orbit coupling (SOC) leads to spin-valley locking and valley (and spin) polarized optical absorptions/emissions^7–12^. On the other hand, the large electron and hole effective masses together with the two-dimensional spatial confinement and the reduced dielectric screening induce the formation of tightly binding excitons and biexcitons. In contrast to conventional semiconductors in which the biexcitons only exist at cryogenic temperature, they can dominate the optical response in TMDs even at room temperature^13–16^, which opens up an opportunity to study a wide range temperature dependence of the exciton and biexciton dynamics.

The SOC in the TMDs leads to spin splitting of both the valence (VB) and the conduction bands (CB) with opposite spin orientation around K and K′ points. The former band arises primarily from the transition metal ${d}_{{x}^{2}-{y}^{2}}$ and *d*_*xy*_ orbitals, showing a splitting of the order of hundreds of meV^17^. Due to its large SOC splitting, optically allowed interband transitions from the upper and lower spin states of the VB are well separated, referring to A and B excitons, respectively. The CB, on the other hand, is predominantly stemmed from ${d}_{{z}^{2}}$ orbital of the metal atom with a small contribution of the chalcogen *p*_*x*_ and *p*_*y*_ orbitals. Since the angular momentum of ${d}_{{z}^{2}}$ orbital is equal to zero, no SOC splitting is expected in the first order approximation. Nevertheless, second-order effects for ${d}_{{z}^{2}}$ orbitals and admixtures of *d*-orbital of M-atoms and *p*-states of X-atoms lead the SOC splitting in the CB to be apparent in spite of being considerably smaller^18^. This latter spin splitting is responsible for the fine structure of both A and B excitons, separating them between bright (spin-aligned) and dark (spin-forbidden) states. According to the ordering of the spin states, the TMDs can be divided into two groups: optically bright monolayers with the aligned spins and optically darkish monolayers with antiparallel spins in the upper valence and lowest conduction subbands. Band structure calculations demonstrate that the ground (first excited) exciton state in the Mo- and W-based TMDs is bright (dark)- and spin-forbidden dark (bright)-state, respectively^19^. Because the photocreated carriers in the photoluminescence measurements at low temperature mainly populate the ground state of the exciton, Mo-based monolayers can efficiently emit light. In contrast, the dark excitons lying in the ground state of the W-based TMDs cannot recombine directly, which induces a significant decrease in the exciton PL intensity when cooling the system. In addition, recent experiments have illustrated that the PL intensity decreases with increasing temperature (4–300 K) by an order of magnitude for MoSe_2_ monolayers, whereas it surprisingly increases an order of magnitude for WSe_2_. Moreover, the experimentally measured degree of valley polarization is quite different in MoX_2_ and WX_2_ systems^20^, indicating that the intervalley scattering depends strongly on the alignment of bright and dark exciton states^21^ which is determined by the CB splitting induced by SOC. Therefore, both the character of the exciton ground state and SOC splitting affect the exciton valley dynamics.

Recently, TMDs ternary alloys (Mo_1−*x*_W_*x*_X_2_) have been synthesized by mixing different transition metals, using either chemical vapour deposition^22^ or conventional low-pressure vapour transport techniques^23,24^. It has been demonstrated that when the tungsten concentration *x* varies continuously from zero to one, the magnitude of SOC^24^, its sign, and the band gap^23,25^ could be engineered by tuning the chemical compositions. It stark contrasts with conventional semiconductor quantum dots where the separation between bright and dark exciton energies can be tuned by size effect, but the character of exciton ground states cannot be changed^26^. Therefore the Mo_1−*x*_W_*x*_X_2_ alloys are ideal platforms to explore mechanisms of exciton valley dynamics, by studying ternary alloys at various compositions. In fact, optical properties and exciton valley dynamics in binary TMDs such as WSe_2_ and MoSe_2_ have been extensively studied^27–31^. However, rather less attention has been paid to the correspondent physical properties of the ternary materials. In particular, the exciton engaged valley dynamics in ternary TMDs remains unknown.

**Figure 1:**
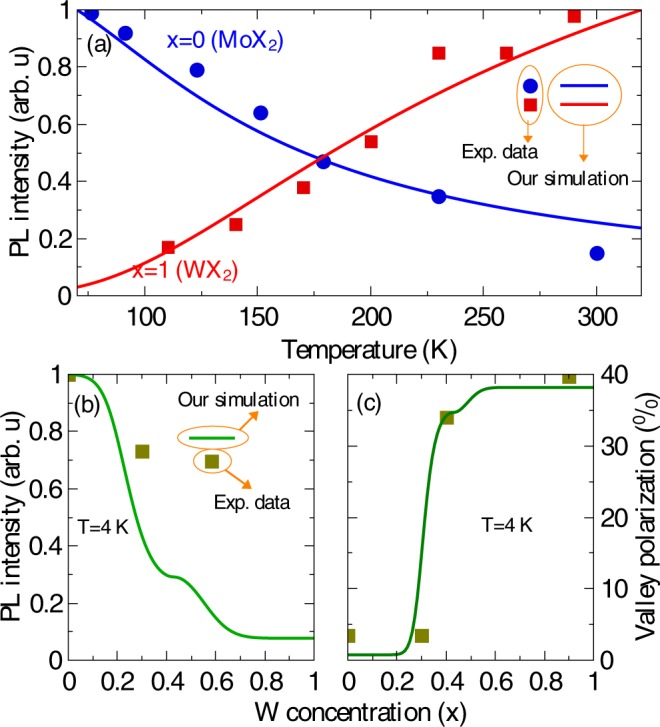
PL intensity and valley polarization of the bright exciton *X*_*b*_ channel of monolayer Mo_1−*x*_W_*x*_X_2_ alloy. (**a**) PL intensity as a function of temperature for the two extreme cases (*x* = 0, MoX_2_, and *x* = 1, WX_2_). Blue and red symbols correspond to experimental data from ref.^32^. (**b**) PL intensity and (**c**) valley polarization degree at *T* = 4 K as a function of W-concentration, solid symbols stand for experimental data (extracted from ref.^24^). Solid curves denote outcome of our calculations in which we consider bright and dark exciton states in both valleys, with intervalley scattering between bright exciton.

In this work, we report a comprehensive theory based on a set of rate equations to investigate the SOC splitting dependent valley (locked with spin) dynamics of bright and dark excitons and of biexcitons in Mo_1−*x*_W_*x*_Se_2_ alloys. For steady-states, we calculate the PL intensity and the valley polarization, taking into account radiative and non-radiative relaxations of excitonic quasiparticles, bright to dark relaxation, dark to bright thermalization, exciton-exciton annihilation, intravalley biexciton formation and intervalley scattering of bright excitons and of biexcitons. A recent work using a similar theoretical approach have studied the PL intensity of excitons and biexcitons in WSe_2_ monolayer in the presence of intervalley momentum-forbidden dark excitons, revealing that phonon-assisted dark-to-bright scatterings lead to an anomalous temperature dependence of PL and to an increase in the valley polarization^19^. Here we investigate how a different kind of dark exciton, i.e., the spin-forbidden intravalley exciton, affects the optical response of TMD monolayers. A complete analysis including both kinds of dark excitons, spin-forbidden and momentum-forbidden, can be found in Supplementary Fig. F1, where we show that the inclusion of indirect excitons does not affect the qualitative conclusions. Furthermore, in this work, we expand the previous analysis by investigating not only binary monolayers but also ternary alloys. The effects of dark excitons can be tuned by changing the W-concentration, which controls the bright-dark intravalley exciton separation via SOC renormalization. To provide suitable propositions to observed phenomena, we have applied our theory to available relevant experimental data in the literature. For instance, recently, Zhang *et al*. performed time-resolved photoluminescence measurements on mechanically exfoliated monolayers of WSe_2_ and MoS_2_. They found that while cooling the system from room temperature down to 110 K, the time-integrated PL intensity of the A exciton in the W-based monolayer, excited by a linear polarized light at low excitation fluence, surprisingly reduces an order of magnitude due to quenching caused by the ground state dark exciton, see red squares in Fig. 1(a). However, in the Mo-based monolayer, an opposite trend is displayed, as indicated by blue circles in Fig. 1(a) ^32^. One can see that our theoretic prediction (solid lines) in the experimental conditions (low pumping intensity regime, i.e., no biexcitons exist) shows very good agreement with experimental data for both WX_2_ and MoX_2_ monolayers. In addition, our theory is applicable not only for the study of PL intensity but also for the analyses of the valley polarization. For example, Wang *et al*. have investigated the impact of tuning the sign and amplitude of the SOC on spin and valley physics in TMD monolayers by circularly polarized optical excitation measurements of Mo_1−*x*_W_*x*_Se_2_ monolayers exfoliated from their bulk counterparts. They found that as the tungsten concentration increases, the PL intensity of the A exciton emission weakens, whereas its valley polarization displays a non-linear increase^24^. Interestingly, the evolution of both PL intensity and valley polarization computed by our theory successfully reproduce experimental measurements, as shown in Fig. 1(b,c). It is worth pointing out that the values of the parameters used in our physical model are not obtained by a specific curve fitting. Instead, they are chosen in such a way that the outcome of our theory can be used to both interpret the available experimental data and also to predict the valley dynamics in experimentally inaccessible TMD monolayers. The outcome of our model demonstrates that the optical properties depend strongly on the SOC splitting in the conduction band, which can be tuned by changing the W-concentration. In this context, the temperature dependent behavior of the PL intensity can be used as a fingerprint to estimate the SOC splitting. Besides, our results also demonstrate that it is possible to grow an optimized alloy by properly selecting its chemical composition *x*, which emits the light with high intensity and strong valley polarization. It paves a way for developing high-performance valleytronic and photo-emitting devices based on the TMDs. Finally, we find that according to the temperature dependence of excitonic valley dynamics, the alloys can be divided into three groups: MoSe_2_-like alloys in which the PL intensity decreases with increasing temperature, WSe_2_ -like alloys where the PL intensity increases with rising temperature, and MoSe_2_ and WSe_2_ mixed alloys in which a transition from the MoSe_2_-like to WSe_2_-like, or vice versa, takes place.

The paper is organized as follows. Sections 2 and 3 are dedicated to describing basic concepts including the three bands model for the TMD alloys and the set of coupled rate equations we propose to describe the exciton dynamics. To gain insights into the contribution of each scattering mechanism, we present our theory starting with a simple physical model, then increasing the complexity in a step by step way by including one additional scattering channel in each step. Section 4 is dedicated to the description of relevant parameters. Our results are presented and discussed in Section 5; in the last section, we present our comments and conclusions.

## Three Bands Model

**Figure 2:**
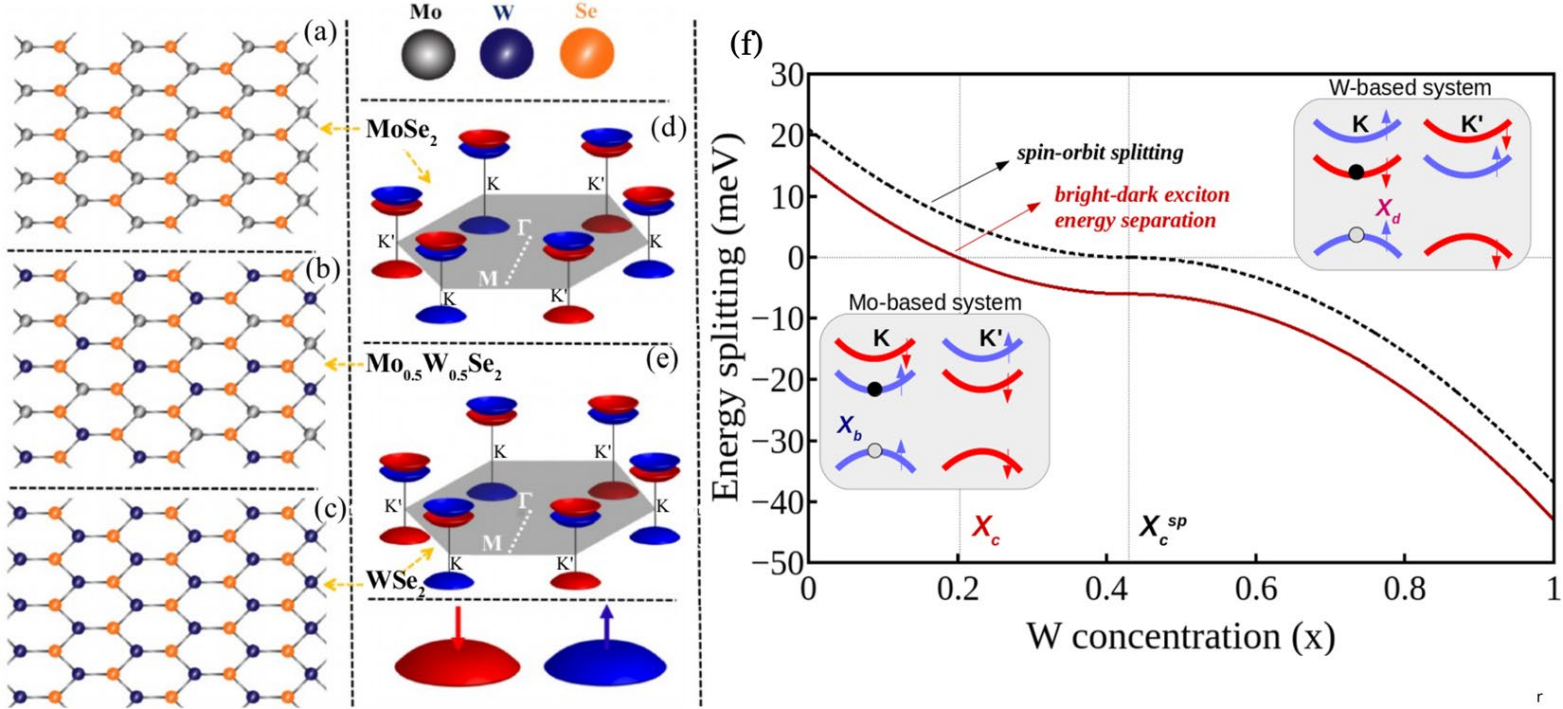
Structure of Mo_1−*x*_W_*x*_Se_2_ monolayer alloy. Top view of the monolayer for (**a**) *x* = 0 (MoSe_2_), (**b**) *x* = 0.5, and (**c**) *x* = 1 (WSe_2_) and Brillouin zone and simplified band dispersion of (**d**) MoSe_2_ and (**e**) WSe_2_. The K and K′ valleys are not equivalent: the conduction and valence bands have opposite spins. (**f**) Bright-dark exciton energy separation and spin-orbit splitting in the conduction bands. *x*_*c*_ corresponds to the critical concentration at which the exciton ground-state changes from bright to dark one and ${x}_{c}^{sp}$ represents the single-particle critical point at which occurs the spin-flip between the two conduction band branches. Insets show schematic representations of the three bands model for Mo-based and W-based compounds around the two valleys. Blue (red) arrows indicate spin up (down) states.

Both binary monolayers (MoX_2_ and WX_2_) and ternary alloys Mo_1−*x*_W_*x*_X_2_ have layered structures, comprised of an inner layer of metal M-atoms sandwiched between two layers of chalcogen X-atoms located on the triangular lattice of alternating hollow sites in a triangular prismatic way; Fig. 2(a–c) show the top view of the crystal structures of MoSe_2_, Mo_0.5_W_0.5_Se_2_, and WSe_2_, respectively. Since ternary alloys possess similar crystal structure to that of binary monolayers, they also have alike electronic structures, i.e., they are direct bandgap semiconductors and the SOC leads to spin splitting between spin-up and spin-down states in both the valence and conduction bands around K and K′ points. As pointed out in the introduction, the spin splitting of the valence band is of the order of hundreds of meV^17^, while it is much smaller in the conduction band, being of the order of tens of meV^18^. In this scenario, the optical signal of intravalley electron-hole bound-states comprised by the two different valence band branches are well separated, comprehending the A and B excitons. In order to gain a deep insight into multiexciton valley dynamics, we focus our attention on the low energy optical response, i.e. A exciton, which can be well described by a three-band model. It is constituted by the higher spin-branch of the valence band and both spin branches of conduction band around the K and K′ points in the Brillouin zone^33^, as schematically represented in Fig. 2(d,e) for MoSe_2_ and WSe_2_, respectively.

The valley dynamics of binary and ternary monolayers depend strongly on the energy alignment and separation of the dark and bright excitons, which determine the thermal equilibrium population in each one of the excitonic states. In the three-band low energy model, the bright and dark exciton energies can be described as

1 $${E}_{B}={E}_{g}+{V}^{d}+{V}^{x}$$

and

2 $${E}_{D}={E}_{g}+{{\rm{\Delta }}}_{SO}^{c}+{V}^{d},$$

respectively, where *E*_*g*_ is the bandgap and ${{\rm{\Delta }}}_{SO}^{c}$ represents the spin-orbit splitting in the conduction bands, which is positive in bright monolayers, such as Mo-based materials, and negative in darkish TMDs, e.g., WX_2_. *V*^*d*^ and *V*^*x*^ are the direct and exchange Coulomb terms, which constitute the electron-hole binding energy. From the Eqs 1 and 2 one notices that the dark-bright energy separation, Δ_*E*_ = *E*_*D*_ − *E*_*B*_ is determined by the SOC in the conduction bands, together with the term stemmed from the repulsive electron-hole exchange interaction. The latter is only non-zero for like-spin transitions (i.e. excitons with total spin along the *z* axis equal to zero)^34,35^. Hence, it does not alter the dark exciton energy, but only shifts the bright exciton energy upwards, due to the different spin configuration of both excitons. Throughout this work, we consider *V*^*x*^ = 6 meV, which is composition independent, as reported in the literature^36^. ${{\rm{\Delta }}}_{SO}^{c}$, on the other hand, can be tuned by changing the W-concentration *x*. To investigate the valley dynamics as a function of *x* in Mo_1−*x*_W_*x*_Se_2_ alloys, we adopt ${{\rm{\Delta }}}_{SO}^{c}(MoS{e}_{2})\simeq 21.0\,{\rm{meV}}$ and ${{\rm{\Delta }}}_{SO}^{c}(WS{e}_{2})\simeq -\,37.0\,{\rm{meV}}$, obtained by density functional theory calculations^37^. As known, the band gap of Mo_1−*x*_W_*x*_Se_2_ alloy monolayer is smaller than the linear combination of that of MoSe_2_ and WSe_2_ due to the so-called bowing effect^23,25^. To properly describe the dependence of spin-orbit splitting ${{\rm{\Delta }}}_{SO}^{c}(x)$ of the alloys on the composition, a non-linear dependence of spin-orbit splitting on *x*, induced by bowing effects, has to be considered. Based on this analysis, we propose that the ${{\rm{\Delta }}}_{SO}^{c}(x)$ is governed by following expression:

3 $${{\rm{\Delta }}}_{SO}^{c}(x)=(1-x)|{{\rm{\Delta }}}_{SO}^{c}(0)|+x|{{\rm{\Delta }}}_{SO}^{c}(1)|-bx(1-x),$$

where the first two terms in the right-hand side correspond to a linear combination of SO splittings of MoSe_2_ and WSe_2_, and the third one describes the bowing effect. From Eq. (3), one can estimate both the single-particle critical concentration ${x}_{c}^{sp}$ passing through which the SOC splitting changes its sign^24^ and the bowing parameter *b* through an analysis of the uniqueness of the solution of the polynomial equation for ${{\rm{\Delta }}}_{SO}^{c}(x={x}_{c}^{sp})=0$, ${x}_{c}^{sp}\in [0,1]$. After a straightforward calculation, we obtain *b* = 113.75 meV and ${x}_{c}^{sp}=0.43$. This single particle critical value matches well with the experimental data, ${x}_{c}^{sp}\simeq 40 \%$^24^, while the bowing parameter for the spin-orbit splitting is of the same order of *b*_*Eg*_ = 140 meV, the bowing parameter for the band gap in the Mo_1−*x*_W_*x*_Se_2_ alloy^25^. The dark-bright energy splitting, ${{\rm{\Delta }}}_{E}={{\rm{\Delta }}}_{SO}^{c}(x)-{V}^{x}$, is plotted in Fig. 2(f) as a function of *x* (solid dark-red line). To comparison purpose, the SO energy splitting in the conduction band is also shown (dotted black line). Note that the exchange interaction shifts the point of excitonic critical concentration to a smaller value when compared with ${x}_{c}^{sp}$. Because dark states in the TMDs possess extremely long radiative and valley-polarization-lifetimes, they are appealing candidates for quantum information. However the dark excitons decouple with light, optical read-out and control of the dark states remain challenging. Therefore, dark exciton brightening in the TMDs has been attracted a lot of attention. Normally, an external magnetic field^36,38,39^ or a magnetic substrate^40^ are exploited to convert optically inactive excitons in optically active one. Nevertheless, owing to a large bright-dark exciton energy separation in the binary TMDs, usually, very high magnetic field or exchange field is required to make dark exciton brightening efficiently. Interestingly, the SOC engineering in the ternary alloys, as shown in Fig. 2(f) sheds a light to figure out this problem. Because there is very small bright-dark exciton energy separation in the alloys with *x* approximate to *x*_*c*_, an accessible magnetic field in the laboratory is big enough to make dark exciton brightening. Therefore, the TMD alloys might move the valleytronics one step further.

With the knowledge of the spin-orbit splitting in the alloys around K and K′ valleys, we are ready to propose a theory to describe the valley dynamics, as described in the next section.

## Theoretical Framework for Multiexciton Valley Dynamics

**Figure 3:**
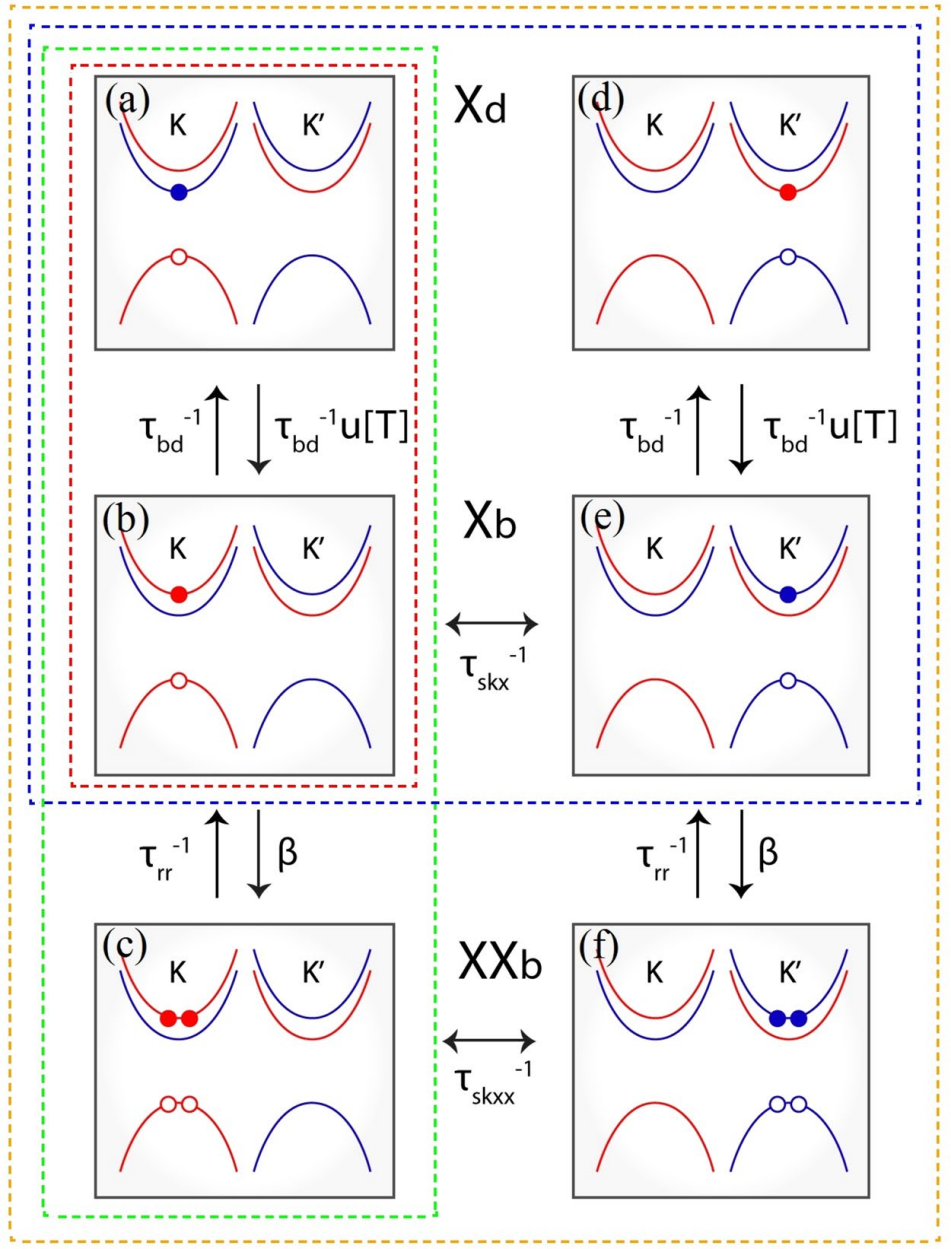
Configuration of bright exciton *X*_*b*_ (${X^{\prime} }_{b}$), intravalley dark exciton *X*_*d*_ (${X^{\prime} }_{d}$), and intravalley bright biexcitons *XX*_*b*_ ($X{X^{\prime} }_{b}$) in the K (K′)-valley of the Mo_1−*x*_W_*x*_Se_2_ monolayer alloy with *x* > *x*_*c*_ (dark exciton ground state). Red (blue) curves stand for spin-up (spin-down) states. Filled (empty) circles represent electrons (holes). The four dotted rectangle enclosing regions represent the four different cases we consider in our work, which include different excitonic channels, as described in Eqs 4 to 7. The alignment of the spins in the two conduction band branches is inverted for *x* < *x*_*c*_ (bright ground state).

**Figure 4:**
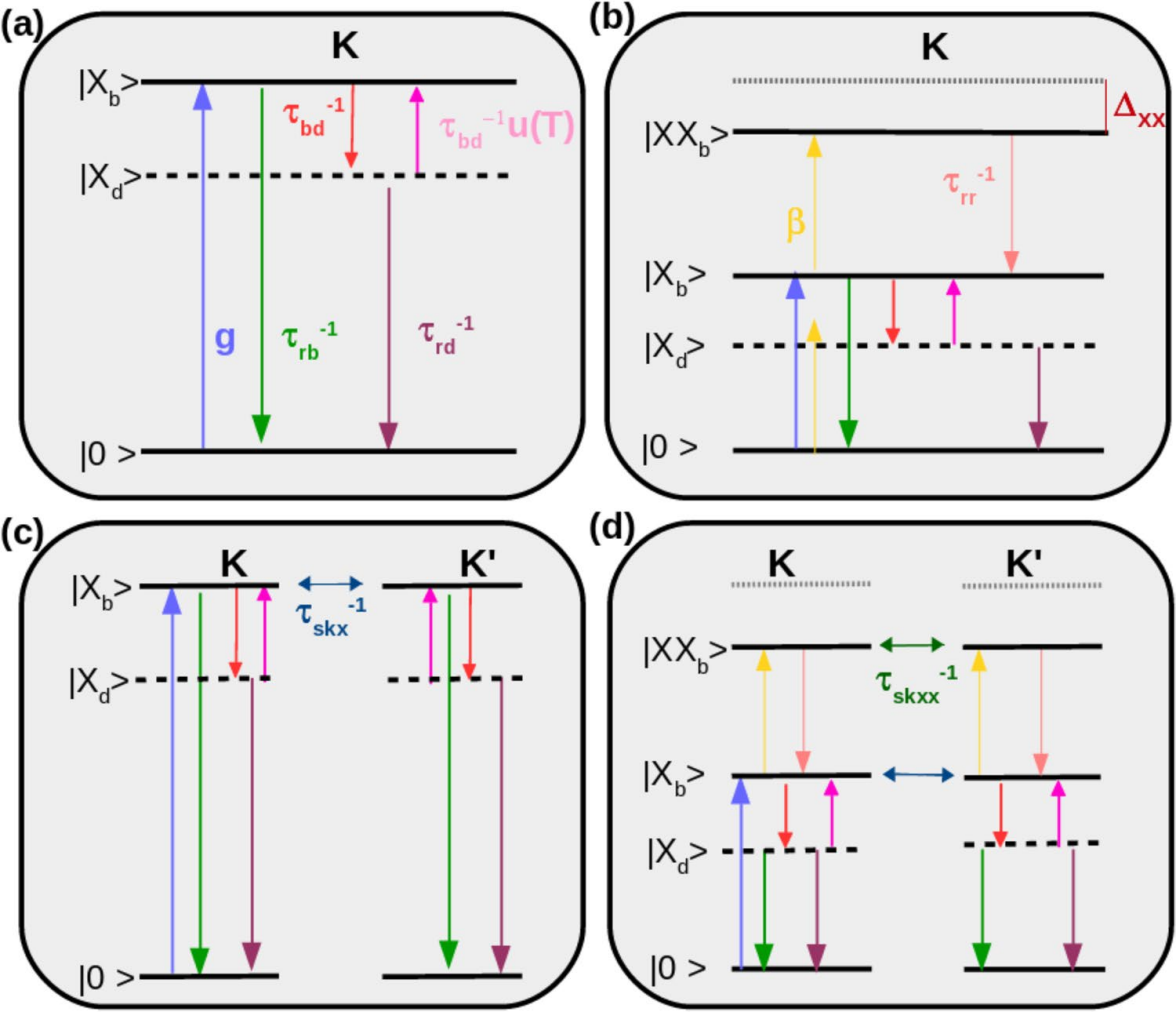
Schematic diagrams of the transitions and scatterings among the ground state |0〉, bright |*X*_*b*_〉 ($|{X^{\prime} }_{b}\rangle$) and dark |*X*_*d*_〉 ($|{X^{\prime} }_{d}\rangle$) exciton state and also bright biexciton states |*XX*_*b*_〉 ($|X{X^{\prime} }_{b}\rangle$) in the K (K’) valley of a Mo_1−*x*_W_*x*_Se_2_ monolayer (*x* > *x*_*c*_, dark exciton ground state) whose directions are indicated by arrows. (**a**) Only the |0〉, |*X*_*b*_〉 and |*X*_*d*_〉 states in the K-valley are considered. *g* stands for the bright exciton generation rate, *τ*_*rb*_ and *τ*_*rd*_ are the bright and dark excitons recombination times, and *τ*_*bd*_ is the bright-dark scattering time. The factor *u*(*T*, *x*) = *exp*(−|Δ*E*(*x*)|/*k*_*b*_*T*) describes the Boltzmann distribution balancing exciton populations between the states |*X*_*b*_〉 and |*X*_*d*_〉, with *k*_*b*_ the Boltzmann constant and *T* the temperature. (**b**) Besides |0〉, |*X*_*b*_〉 and |*X*_*d*_〉 states, a bright biexciton state in the K-valley is also included. In this case *τ*_*rbb*_ represents the biexciton recombination time, *β* corresponds to the exciton-to-biexciton transition rate and Δ_*xx*_ stands for the biexciton binding energy, i.e. ${E}_{X{X}_{b}}=2{E}_{{X}_{b}}-{{\rm{\Delta }}}_{xx}$. (**c**) Intravalley scatterings in the K-valley as shown in (**a**) and in the valley K′ and intervalley scattering between |*X*_*b*_〉 and $|{X^{\prime} }_{b}\rangle$ (with a scattering rate given by ${\tau }_{skx}^{-1}$) are included. In this case, the index ′ stands for the states in the K′-valley, where we consider a similar behavior for the transitions and scatterings since the states of two valleys are related by time-reversal symmetry. We do not include, though, a bright exciton generation rate in the latter valley, because we consider that the system is excited by right circularly polarized light fields (*σ*^+^); since the valley selective transition rule depends on the helicity of light fields, the optical excitation only occurs in the K-valley. (**d**) In the most complete case, we include both the bright biexciton states and the intervalley scattering between bright excitons and bright biexcitons. The latter happens with a rate given by ${\tau }_{skxx}^{-1}$.

There are several different excitonic states in TMD monolayers, such as bright and dark excitons and biexcitons, which can be in either K or K′ valleys. In order to understand the multiexciton valley dynamics as a function of both temperature and tungsten concentration, the recombinations and scatterings among these quasiparticles, including intravalley and intervalley scatterings, are taken into account in our model. To reveal the effect of each scattering channel, we split our study into four different cases which are schematically represented in Figs 3 and 4. In these figures, *X*_*b*_ and *X*_*d*_ denote bright and dark excitons, and *XX*_*b*_ represents bright biexciton in the K-valley. ${X^{\prime} }_{b}$, ${X^{\prime} }_{d}$, and $X{X^{\prime} }_{b}$ stand for the corresponding quasiparticles in the K′-valley (hereafter, apostrophe ′ stands for the states in K′-valley excitonic channels). It is worth recalling that both figures represent alloys with *x* > *x*_*c*_ (dark exciton ground state). For the case in which *x* < *x*_*c*_ one has to invert the positions of the |*X*_*b*_〉 and |*X*_*d*_〉 state in both valleys. The set of equations we use to describe each one of the processes illustrated in Fig. 4 are described in the following; we carry out our study according to the order of complexity.

### (a) Intravalley bright and dark exciton dynamics

In the simplest case [Fig. 4(a)], we investigate the exciton recombination kinetics involving scatterings between intravalley bright and dark states. For *x* < *x*_*c*_, the ground state is optically active (bright exciton), then the scattering rate from bright to dark state is described by $\frac{1}{{\tau }_{bd}}u(T,x)$, while the scattering from dark to bright state is given by $\frac{1}{{\tau }_{bd}}$. In contrast, the ground state is optically inactive for *x* > *x*_*c*_, then the scattering rates are inverted, i.e. $\frac{1}{{\tau }_{bd}}$ for the exciton scattered from bright to dark states and $\frac{1}{{\tau }_{bd}}u(T,x)$ for the exciton scattered in the opposite direction. Here *u*(*T*, *x*) = *exp*(−|Δ*E*(*x*)|/*k*_*b*_*T*) is the Boltzmann distribution function which balances exciton population in the bright and dark states, reflecting the presence of the energy barrier Δ*E* due to dark-bright energy splitting (see Fig. 2(f) and Eq. 3), and *k*_*b*_ is the Boltzmann constant. In a three-level model, the exciton dynamics, as illustrated by the red rectangular scheme in Figs 3 and 4(a), can be described by two coupled rate equations:

4 $$\begin{array}{rcl}\frac{d{n}_{b}}{dt} & = & g-\frac{{n}_{b}}{{\tau }_{rb}}-\frac{{n}_{b}}{{\tau }_{bd}}{{\rm{\Theta }}}_{({x}_{c}-x)}+\frac{{n}_{d}}{{\tau }_{bd}}{{\rm{\Theta }}}_{(x-{x}_{c})}\\ \frac{d{n}_{d}}{dt} & = & -\frac{{n}_{d}}{{\tau }_{rd}}+\frac{{n}_{b}}{{\tau }_{bd}}{{\rm{\Theta }}}_{({x}_{c}-x)}-\frac{{n}_{d}}{{\tau }_{bd}}{{\rm{\Theta }}}_{(x-{x}_{c})},\end{array}$$

where the modified step function ${{\rm{\Theta }}}_{(x-{x}_{c})}$ is defined by

$${{\rm{\Theta }}}_{(x-{x}_{0})}=\{\begin{array}{ll}u(T,x) & {\rm{if}}\,x > {x}_{0};\\ 1 & {\rm{if}}\,x < {x}_{0}.\end{array}$$

Here *n*_*b*_ and *n*_*d*_ are concentrations of bright *X*_*b*_ and dark *X*_*d*_ excitons in the K-valley, *g* stands for the bright exciton generation rate, *τ*_*rb*_ and *τ*_*rd*_ are the bright and dark excitons recombination time, and *τ*_*bd*_ is the bright-dark scattering time.

### (b) Dynamics of intravalley bright and dark excitons, and biexcitons

When the laser excitation intensity increases, more excitons are created and strong Coulomb interactions favor the Auger-type exciton-exciton annihilation process, giving rise to the formation of bright biexcitons (states consisting of two excitons) whose population depends quadratically on the exciton concentration. It is known that experimentally detecting spectroscopic features of biexcitons is extremely challenging in conventional semiconductors due to the very small biexciton binding energy. However, experimental evidence of the biexciton in the TMDs have been reported even at low-excitation power^41^ and at room temperature^42^. The decay of biexcitons leads to the generation of excitons. Since we use circularly polarized light to create excitons, the number of intravalley biexcitons dominate over intervalley ones. Thus, throughout this work we consider that two bright excitons can bind together forming an intravalley biexciton, as illustrated in Fig. 4(b). We also note that very recently a series of experimental works have reported the observation of a new kind of biexciton, composed of one bright and one dark exciton^43–48^. However, we have observed that the inclusion of the fine structure of biexcitons slightly alters the optical response of bright intravalley excitons in the TMD monolayer alloy (see Supplementary Material, Sec. II).

The dynamics of intravalley bright and dark excitons and bright-bright biexcitons is described by the following set of coupled rate equations:

5 $$\begin{array}{rcl}\frac{d{n}_{b}}{dt} & = & g-\frac{{n}_{b}}{{\tau }_{rb}}-\frac{{n}_{b}}{{\tau }_{bd}}{{\rm{\Theta }}}_{({x}_{c}-x)}+\frac{{n}_{d}}{{\tau }_{bd}}{{\rm{\Theta }}}_{(x-{x}_{c})}-2\beta {n}_{b}^{2}+\frac{{n}_{bb}}{{\tau }_{rbb}}\\ \frac{d{n}_{d}}{dt} & = & -\frac{{n}_{d}}{{\tau }_{rd}}+\frac{{n}_{b}}{{\tau }_{bd}}{{\rm{\Theta }}}_{({x}_{c}-x)}-\frac{{n}_{d}}{{\tau }_{bd}}{{\rm{\Theta }}}_{(x-{x}_{c})}\\ \frac{{n}_{bb}}{dt} & = & -\frac{{n}_{bb}}{{\tau }_{rbb}}+\beta {n}_{b}^{2},\end{array}$$

where n_*bb*_ is the concentration of bright biexcitons in the K-valley and *β* corresponds to the exciton-to-biexciton transition rate, which depends on the laser power. In a reversed process, we consider that the decay of a biexciton with a rate of ${\tau }_{rbb}^{-1}$ leads to the generation of excitons.

### (c) Valley dynamics of bright and dark excitons

Due to the combined effect of inversion symmetry breaking and strong spin-orbit interaction in TMD monolayers, interband transitions are governed by chiral selection rules which allows efficient optical initialization of an electron-hole pair in a specific valley (either K or K′) in momentum space. More specifically, TMD monolayers endure valley dependent optical selection rules, that is, the K- (K′-) valley can be selectively excited by *σ*^+^ (*σ*^−^) circularly polarized photons, which results in the emitted light with the same helicity of the excitation laser. In this scenario, one might expect light emission in TMD monolayers with 100% of valley polarization. Polarization-resolved PL experiments have shown, however, that the degree of valley polarization is very low, especially for Mo-based TMDs^20^, indicating that there are relevant intervalley scatterings in these materials. We consider that the intervalley scattering is mediated by the exchange Coulomb interaction^49^, which acts as an effective in-plane magnetic field on the valley degree of freedom. The precession of the valley pseudospin around this effective field leads to the intervalley scattering. The intervalley scattering could also take place through longitudinal acoustic phonons^50^. However, since the exchange-mediated scattering is a zero-energy process^51^, it dominates the valley scattering process due to the valley degeneracy ensured by time-reversal symmetry. Therefore, to properly describe the dynamics of exciton population in a particular valley (K/K′), the terms related to intervalley scattering should be incorporated in the rate equations. With illustration purpose, we start with the systems comprising of bright and dark exciton states in both K and K′ valleys, assuming that there are intervalley scattering between bright excitons, as schematically represented in Fig. 4(c). The corresponding rate equations are:

6 $$\begin{array}{rcl}\frac{d{n}_{b}}{dt} & = & g-\frac{{n}_{b}}{{\tau }_{rb}}-\frac{{n}_{b}}{{\tau }_{skx}}+\frac{{n}_{b^{\prime} }}{{\tau }_{skx}}-\frac{{n}_{b}}{{\tau }_{bd}}{{\rm{\Theta }}}_{({x}_{c}-x)}+\frac{{n}_{d}}{{\tau }_{bd}}{{\rm{\Theta }}}_{(x-{x}_{c})}\\ \frac{d{n}_{b^{\prime} }}{dt} & = & g^{\prime} -\frac{{n}_{b^{\prime} }}{{\tau }_{rb}}-\frac{{n}_{b^{\prime} }}{{\tau }_{skx}}+\frac{{n}_{b}}{{\tau }_{skx}}-\frac{{n}_{b^{\prime} }}{{\tau }_{bd}}{{\rm{\Theta }}}_{({x}_{c}-x)}+\frac{{n}_{d^{\prime} }}{{\tau }_{bd}}{{\rm{\Theta }}}_{(x-{x}_{c})}\\ \frac{d{n}_{d}}{dt} & = & -\frac{{n}_{d}}{{\tau }_{rd}}-\frac{{n}_{d}}{{\tau }_{bd}}{{\rm{\Theta }}}_{(x-{x}_{c})}+\frac{{n}_{b}}{{\tau }_{bd}}{{\rm{\Theta }}}_{({x}_{c}-x)}\\ \frac{d{n}_{d^{\prime} }}{dt} & = & -\frac{{n}_{d^{\prime} }}{{\tau }_{rd}}-\frac{{n}_{d^{\prime} }}{{\tau }_{bd}}{{\rm{\Theta }}}_{(x-{x}_{c})}+\frac{{n}_{b^{\prime} }}{{\tau }_{bd}}{{\rm{\Theta }}}_{({x}_{c}-x)}.\end{array}$$

Here *τ*_*skx*_ denotes the intervalley scattering time between bright excitons in two different valleys. In the equations above, the index′ stands for the states in the K′-valley. Since the two valleys are related by time-reversal symmetry, we assume that the transition and scattering rates are the same in K and K′ valleys. It is worthy to point out that the exchange interaction cannot scatter spin-forbidden dark exciton from one valley to the other^20,52^, and even though there are other mechanisms which causes an intervalley scattering between dark states, the relevant time is about one order of magnitude larger than that of bright states^53^. Thus, the intervalley scattering between two dark states is not considered.

### (d) Valley dynamics of exciton (bright and dark) and biexciton states

After describing intravalley dynamics of multiexcitons and valley dynamics of bright excitons, we are ready to investigate valley dynamics in more complex systems which involve both intravalley scatterings among bright exciton, dark exciton and biexciton states in both K- and K′-valleys and intervalley scatterings between bright excitons and between bright biexcitons, with a rate given by ${\tau }_{skxx}^{-1}$. All these processes are schematically represented in Fig. 4(d) and can be described by the following set of equations:

7 $$\begin{array}{rcl}\frac{d{n}_{b}}{dt} & = & g-\frac{{n}_{b}}{{\tau }_{rb}}-\frac{{n}_{b}}{{\tau }_{skx}}+\frac{{n}_{b^{\prime} }}{{\tau }_{skx}}-\frac{{n}_{b}}{{\tau }_{bd}}{{\rm{\Theta }}}_{({x}_{c}-x)}\\  &  & +\,\frac{{n}_{d}}{{\tau }_{bd}}{{\rm{\Theta }}}_{(x-{x}_{c})}+\frac{{n}_{bb}}{{\tau }_{rbb}}-2\beta {n}_{b}^{2}\\ \frac{d{n}_{b^{\prime} }}{dt} & = & g^{\prime} -\frac{{n}_{b^{\prime} }}{{\tau }_{rb}}-\frac{{n}_{b^{\prime} }}{{\tau }_{skx}}+\frac{{n}_{b}}{{\tau }_{skx}}-\frac{{n}_{b^{\prime} }}{{\tau }_{bd}}{{\rm{\Theta }}}_{({x}_{c}-x)}\\  &  & +\,\frac{{n}_{d^{\prime} }}{{\tau }_{bd}}{{\rm{\Theta }}}_{(x-{x}_{c})}+\frac{{n}_{b^{\prime} b^{\prime} }}{{\tau }_{rbb}}-2\beta {n}_{b^{\prime} }^{2}\\ \frac{d{n}_{d}}{dt} & = & -\,\frac{{n}_{d}}{{\tau }_{rd}}-\frac{{n}_{d}}{{\tau }_{bd}}{{\rm{\Theta }}}_{(x-{x}_{c})}+\frac{{n}_{b}}{{\tau }_{bd}}{{\rm{\Theta }}}_{({x}_{c}-x)}\\ \frac{d{n}_{d^{\prime} }}{dt} & = & -\,\frac{{n}_{d^{\prime} }}{{\tau }_{rd}}-\frac{{n}_{d^{\prime} }}{{\tau }_{bd}}{{\rm{\Theta }}}_{(x-{x}_{c})}+\frac{{n}_{b^{\prime} }}{{\tau }_{bd}}{{\rm{\Theta }}}_{({x}_{c}-x)}\\ \frac{d{n}_{bb}}{dt} & = & -\,\frac{{n}_{bb}}{{\tau }_{skxx}}-\frac{{n}_{bb}}{{\tau }_{rbb}}+\frac{{n}_{b^{\prime} b^{\prime} }}{{\tau }_{skxx}}+\beta {n}_{b}^{2}\\ \frac{d{n}_{b^{\prime} b^{\prime} }}{dt} & = & -\,\frac{{n}_{b^{\prime} b^{\prime} }}{{\tau }_{skxx}}-\frac{{n}_{b^{\prime} b^{\prime} }}{{\tau }_{rbb}}+\frac{{n}_{bb}}{{\tau }_{skxx}}+\beta {n}_{b^{\prime} }^{2}\end{array}$$

With the proposed coupled rate equations for the exciton and biexciton dynamics, we can calculate two experimentally observable quantities: the PL intensity and the valley polarization. Since the light emission depends on both the transition rate and the population of the corresponding state, we compute the PL intensity for each individual excitonic channel via *I*_*j*_ = *n*_*j*_/*τ*_*rj*_^33,54^, where *n*_*j*_ and *τ*_*rj*_ denote the concentration and the recombination time of the excitonic channel *j*, here *j* = *b*/*b*′, *d*/*d*′, and *bb*/*b*′*b*′ corresponding to bright, dark and biexciton states in the K/K′ valley. To compute the steady-state PL intensity, we solve the coupled rate Eqs 4–7, setting the left-hand sides of them to be equal to zero, i.e., *dn*_*j*_/*dt* = 0. Unless specified, we assume that the TMD alloys are pumped by a circularly polarized light field with *σ*^+^ polarization. In this case, the optical absorption only occurs in the K-valley, i.e., *g* = 5.35 10^−6^ cm^−2^ s^−1^ and *g*′ = 0. However, owing to the intervalley scatterings, optical emissions can arise in both K and K′ valleys, which could be detected by *σ*^+^ and *σ*^−^, respectively. The degree of valley polarization is proportional to the difference in PL intensities between *σ*^+^ and *σ*^−^ emissions for the system excited by *σ*^+^ polarized light, and is defined by $V{P}_{j}=\frac{{I}_{j}(K)-{I}_{j}(K^{\prime} )}{{I}_{j}(K)+{I}_{j}(K^{\prime} )}$. Owing to the time-reversal symmetry, the *σ*^+^ emission intensity of an exciton in the K-valley excited by *σ*^+^ light is equal to that of *σ*^−^ emission in the K′-valley pumped by *σ*^−^ laser. For simplicity but without loss of generality, we only show the PL intensities of the excitonic emissions in the K-valley in the forthcoming figures, unless otherwise stated specifically.

## Relevant Parameters

Throughout this work, we consider that relaxation rates and scattering times do not depend on the chemical composition *x* of the alloys^20,55^. Accordingly, the composition dependence of the excitonic valley dynamics is manifested by the magnitude and the sign of the SOC splitting Δ*E*(*x*), that separates dark and bright excitons, as defined in Eq. 3 and represented is Fig. 2(f). In addition, we consider that the radiative bright exciton recombination time depends linearly on the temperature *T*, i.e., *τ*_*rb*_ = *αT*, with *α* = 10 ps/K, as observed in 2D semiconductor quantum wells^56,57^ and TMD monolayers^58^. The observed increase of the exciton linewidth (and the corresponding rise of the exciton recombination time) is typically ascribed to scattering with acoustic and optical phonons within the valleys at low^59^ and high temperatures^60,61^. We also suppose that the biexcitons have the same decay time as the bright excitons, i.e., *τ*_*rbb*_ = *τ*_*rb*_, but the dark excitons have a longer lifetime, *τ*_*rd*_ = 1.0 ns, because they decay non-radiatively^58^. The scattering time between the bright and dark states, *τ*_*bd*_ = 1 ps, is chosen in such a way that it is comparable to the bright exciton recombination time at low temperature, but shorter at high temperatures, as reported in the literature^20^. Furthermore, we adopt fast intervalley-scattering times *τ*_*skx*_ = 0.01 ps and *τ*_*skxx*_ = 0.02 ps, for bright excitons and bright biexcitons, respectively. The scattering time orders of magnitude smaller than the exciton lifetime can be attributed to the efficient exchange-driving intervalley scattering^20,52,62^.

Using these parameters, our calculated valley polarization is in coincidence with the experimental data of ref.^24^, as shown in Fig. 1(c). Finally, we consider a power dependent exciton-to-biexciton transition rate *β* = *β*_0_/(1 + *P*/*P*_0_)^13^, with the laser power P = 1.0 kW/cm^2^ (unless otherwise stated), *P*_0_ = 10.2 kW cm^−2^ and *β*_0_ = 36.0 nm^2^ ps^−1^. The parameters adopted in our simulations [Eqs 4 to 7] are summarized in Table 1.

**Table 1:** Relevant parameters [Eqs 4 to 7] used in our simulations^13,20,24,42,58^.

*τ*_*rb*_ = 10 *T* ps/K	*τ*_*rbb*_ = *τ*_*rb*_	*τ*_*rd*_ = 1.0 ns
*τ*_*skx*_ = 0.01 ps	*τ*_*skxx*_ = 0.02 ps	*τ*_*bd*_ = 1.0 ps
*P* = 1.0 kW/cm^2^	*P*_0_ = 10.2 kW cm^−2^	*β*_0_ = 36.0 nm^2^ ps^−1^
*g*′ = 0.0	*g* = 5.35 10^−6^ cm^−2^ s^−1^	

To verify the validity of our model and assure that the values of parameters in the rate equations are properly chosen, we make a comparison between our calculated PL intensity (solid lines) and experimental data (blue circles and red squares) of ref.^32^ for the two extreme cases, *x* = 0 and *x* = 1, as shown in Fig. 1(a). The scatterings between intravalley bright- and dark- excitons in either K- or K′-valley, and intervalley scatterings between the bright excitons [see Fig. 4(c) and Eq. 6] are taken into account. Note that the PL intensity of WX_2_ decreases with decreasing temperature because of a lower-lying dark state that quenches the emission of the bright excitons. Opposite temperature dependence is observed in the MoX_2_. This behavior is the same as that of normal semiconductors, stemmed from increased non-radiative recombinations at high temperatures^63^. We also extend our comparison to a more general case in which the tungsten concentration *x* of Mo_1−*x*_W_*x*_Se_2_ alloys varies from zero to one. In this case, not only the PL intensity but also the valley polarization of bright-exciton emission were analyzed (shown by solid lines in Fig. 1(b,c), while symbols represent experimental data of ref.^24^). As *x* increases from zero, the physical properties of the alloy can be significantly tuned: the valley polarization is strongly enhanced, nevertheless accompanied by a reduction of the PL intensity. Clearly, the model here proposed was capable of successfully reproducing the overall behavior of experimental data.

## Results and Discussion

**Figure 5:**
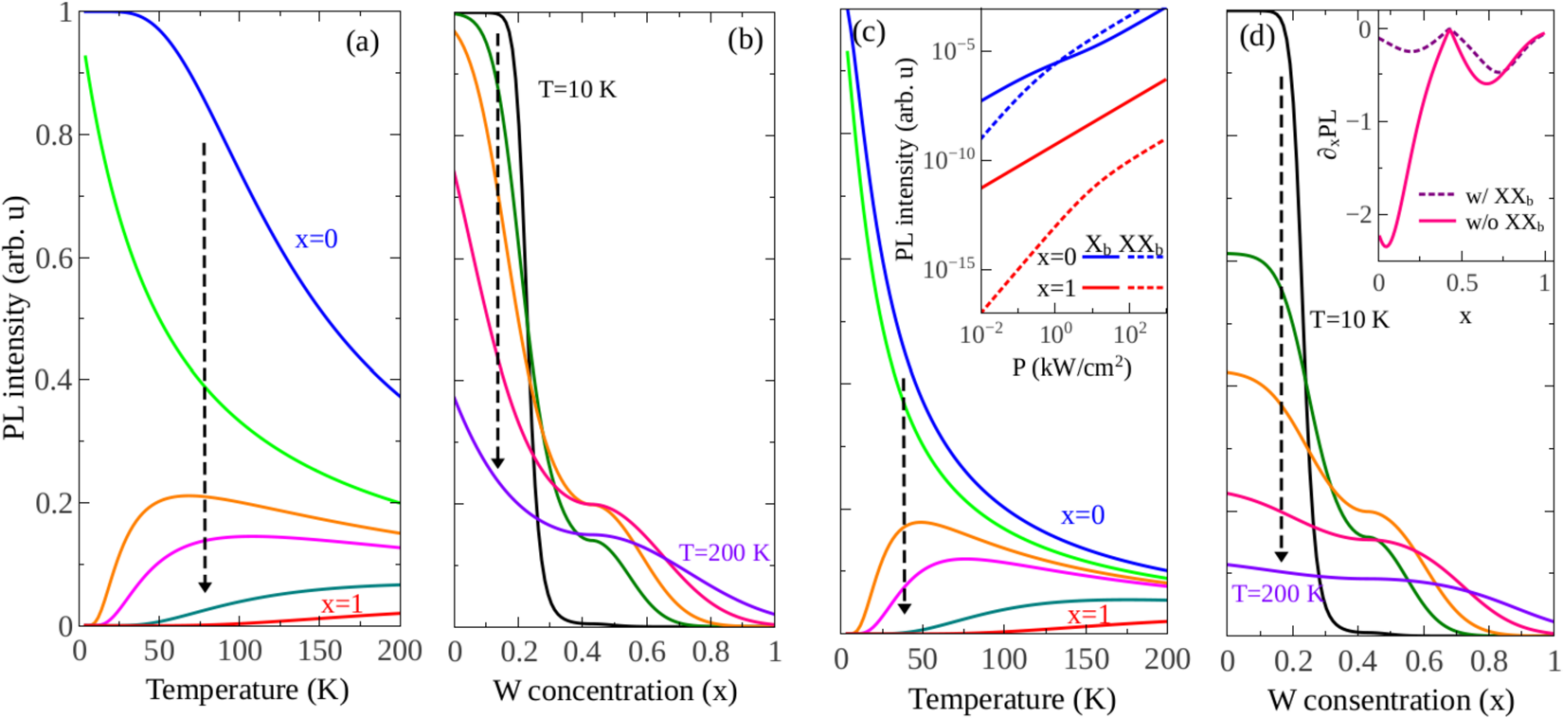
PL intensity of the bright exciton channel *I*_*b*_ = *n*_*b*_/*τ*_*rb*_ in monolayer Mo_1−*x*_W_*x*_Se_2_ alloy excited with a right circularly polarized continuous wave laser. (**a** and **c**) PL intensity as a function of temperature obtained with different tungsten concentrations *x* whose value increases from 0.0 to 1.0 by a step of 0.2 as indicated by the vertical arrow. (**b** and **d**) PL intensity as a function of *x* for different values of temperature from *T* = 10 to 200 K. The vertical arrow gives the direction along with temperature rises. In (**a** and **b**) we assume that only intravalley bright and dark excitons exist and no intervalley scatterings take place. In (**c** and **d**) we include intravalley biexciton channels. For all panels, we have normalized the PL data in such a way that the larger PL intensity is equal to one. Inset in (**c**) PL intensity of the bright exciton *X*_*b*_ (solid lines) and bright biexciton *XX*_*b*_ (dotted lines) emissions as a function of the laser power *P* for *x* = 0 (blue curves) and 1.0 (red curves) at *T* = 10 K. We assume that all the excitonic quasiparticles are in a single valley. Inset in (**d**) Derivative of the bright exciton PL intensity with respect to W-concentration for an intermediate temperature *T* = 100 K. The solid pink line corresponds to the results computed for the system without the biexciton channel (Eq. 4), while the dotted purple line represents the case in which we include biexcitons (Eq. 5).

We start analyzing the simplest case, where only intravalley bright and dark exciton scatterings play a role (Fig. 4(a) and Eq. 4). Figure 5(a,b) displays the PL intensity of bright-exciton in monolayer Mo_1−*x*_W_*x*_Se_2_ alloys as a function of temperature (W-concentration) for different W-concentrations (temperatures). Three different behaviors of the PL intensity on temperature are found. For *x* < *x*_*c*_ = 0.2, the PL intensity decreases with rising temperature [see the upper two curves in Fig. 5(a)], whereas an opposite behavior is found for large *x*, as shown in the lowest two curves. For intermediate W-concentration, however, a non-monotonic temperature dependence emerges (see the orange and pink curves), in very good agreement with recent temperature-dependent PL measurement of the monolayer Mo_0.5_W_0.5_Se_1.9_ alloy^64^. Hence, for low and high *W*-concentrations, the Mo_1−*x*_W_*x*_Se_2_ ternary monolayer alloys show MoSe_2_ and WSe_2_ like behaviors, respectively, while a transition from W-based to Mo-based behaviors occurs at intermediate *x* values.

In addition, Fig. 5(b) shows that, although the PL intensity exhibits an overall decrease as *x* increases, we can identify three regimes as a function tungsten concentration: usual temperature dependent regime (I) in which the PL intensity decreases with increasing temperature, phase transition region (II) and anomalous temperature dependence region (III) where the PL intensity increases with rising temperature. In region II, the crossovers between different PL curves occur, which indicates the switch of the character from the bright to dark exciton ground state. Moreover, the dependence of the PL intensity on *x* becomes weaker as the temperature rises. The underlying physics is as follows. In region I (0 ≤ *x* < *x*_*c*_), the bright-state is the ground state, as illustrated in Fig. 2(f). At low-temperature, the photocreated excitons largely populate the ground state and then efficiently recombine to emit light. As observed in conventional semiconductors, the PL intensity in these alloys decreases with rising temperature due to thermally activated scatterings. In contrast, in region III (*x* > *x*_*c*_), the ground state becomes dark, where excitons cannot recombine directly, requiring thus disorder and/or phonons to break the spin selection rule. Then the PL emission is quenched at low temperature due to an accumulation of excitons in this state. When the sample is heated up, the occupation probability of the bright state increases while the nonradiative rate remains almost constant. Therefore the PL signal is intensified. Finally, in region II (*x* ≈ *x*_*c*_), the bright-dark excitons separation is quite small, leading the thermal activation to be very effective. Even in the case of a dark ground state, very small thermal energy is enough to excite the excitons from dark to bright state. Then the PL exhibits a nonmonotonic temperature dependence, shown by the orange curve in Fig. 5(a).

As previously described, in the regime of high excitation intensity, the Auger-type exciton-exciton (collision) annihilation process gives rise to the formation of bright biexcitons. In Fig. 5(c,d) we show how this extra excitonic channel affects the intravalley bright exciton recombination kinetics in the alloys displayed in Fig. 5(a,b). The inset of Fig. 5(c) shows the PL intensity of the X_*b*_ and XX_*b*_ as a function of pumping power for MoSe_2_ (blue lines) and WSe_2_ (red lines) at low temperature, *T* = 10 K. Note that the PL intensity of either X_*b*_ or XX_*b*_ in MoSe_2_ is larger than its counterpart in WSe_2_. In addition, in the former, the exciton dominates the optical process in low excitation power range, whereas the PL intensity of the XX_*b*_ surpasses that of exciton at high fluences. In contrast, the PL intensity of the exciton emission in WSe_2_ is always larger than that of the biexciton in the interval of laser power from 0.01 to 100 kW/cm^2^. In the following, we choose *P* = 1.0 kW/cm^2^ in our calculation. Figure 5(c,d) is a similar plot to Fig. 5(a,b), except for the additional biexciton channel. A comparison between both figures shows that, for bright alloys (*x* < *x*_*c*_), the inclusion of the biexciton channel leads to a steeper decrease of the bright exciton PL intensity with rising temperature, which weakens the dependence of the PL intensity on the tungsten concentration, especially in the regime of high temperatures. As a consequence, the curves corresponding to *x* = 0 and *x* = 0.2 get closer in Fig. 5(c). This behavior leads to the conclusion that the presence of biexciton stabilizes the exciton emission against the variation of the W-concentration. More precisely, the presence of the biexciton opens an extra scattering channel for the bright exciton aside from the scattering between the bright and dark excitons. Both of them tend to weaken the bright exciton emission. The former depends neither on the temperature nor on the chemical composition of the alloys, nevertheless the latter depends strongly on the W-concentration and is enhanced by rising temperature. Then in the low-temperature regime, the reduction of exciton PL intensity mainly stems from the biexciton scattering channel. With increasing temperature, however, both scattering channels play a role. As expected, because of lower biexciton density and dark exciton ground state, this effect becomes less pronounced in the alloys with *x* > *x*_*c*_. More details of the effect of the biexciton channel on the bright exciton PL intensity can be seen in the inset of Fig. 5(d), which displays the derivative of the bright exciton PL intensity in the K valley, *I*_*b*_, with respect to *x* at an intermediate temperature *T* = 100 K. Solid pink line computed from Eq. 4 corresponds to the system in the regime of low excitation fluence, i.e., without the biexciton channel, while the dotted purple line represents the case in which the biexciton channel (Eq. 5) is also included aside from the bright and dark exciton channels. Despite the lack of a direct physical meaning, this derivative helps one to clearly see the difference in the W-concentration dependence of the bright exciton PL intensity in the presence and absence of the biexciton channels. Notice that for bright alloys (*x* < *x*_*c*_) the dotted purple curve only slightly depends on *x* (∂_*x*_*PL* ≈ 0), indicating that the biexciton channel weakens the dependence of the bright exciton PL intensity on the W-concentration. Nevertheless, the derivative changes dramatically in darkish alloys due to the bright-darkish transition.

Let us now analyze the valley polarization of excitonic emissions in the alloys by switching on intervalley scatterings. In the simplest scenario, in which only bright excitons exist, the degree of valley polarization at the steady state condition depends on the ratio between the exciton lifetime *τ*_*rb*_ and the intervalley scattering time *τ*_*skx*_, $VP=1/(1+2\frac{{\tau }_{rb}}{{\tau }_{skx}})$^55^; a high degree of valley polarization is achieved when the intervalley scattering time is larger than the exciton recombination time.

**Figure 6:**
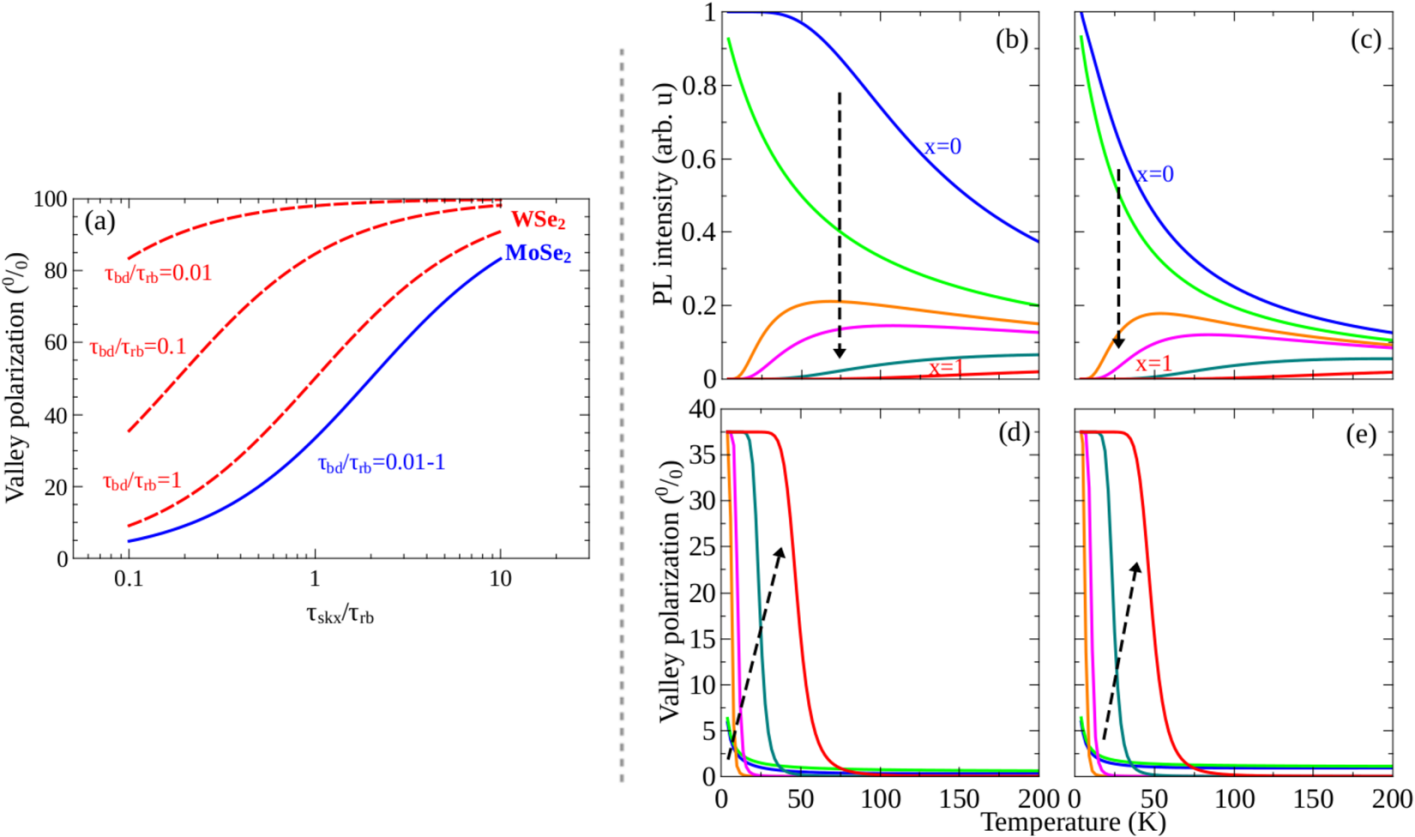
PL intensity and valley polarization of the bright exciton *X*_*b*_ channel of monolayer Mo_1−*x*_W_*x*_Se_2_ alloy excited by a right circularly polarized continuous wave laser. (**a**) Valley polarization of bright excitons *X*_*b*_ PL as a function of the intervalley scattering time *τ*_*skx*_ for WSe_2_ (red dashed lines) and MoSe_2_ (blue solid lines) for three different values (0.01*τ*_*rb*_, 0.1*τ*_*rb*_, 1.0*τ*_*rb*_) of the bright-dark exciton scattering time *τ*_*bd*_. *τ*_*rb*_ = 100 ps is exciton radiation. Calculation performed for *T* = 10 K. (**b**–**e**) PL intensity and valley polarization as a function of temperature obtained with different tungsten concentrations. The results displayed on panels (b and d) were obtained by accounting for *X*_*b*_, *X*_*d*_, ${X^{\prime} }_{b}$ and ${X^{\prime} }_{d}$ with intravalley scattering channels in both K and K’ valleys and also intervalley scattering between bright excitons. Panels (c and e) correspond to the case in which we add bright biexciton channels in both valleys. The arrows indicate the direction of increasing *x*. Panels (b and c) were independently normalized in such a way that the larger PL intensity, in each one, is equal to unit.

A more complex system emerges when we consider both bright and dark excitons. The exciton dynamics are represented in Fig. 4(c) and described by Eq. 6. Figure 6(a) shows the calculated degree of polarization as a function of the intervalley scattering time *τ*_*skx*_ for WSe_2_ (red dashed lines) and MoSe_2_ (blue solid line) TMDs, obtained for three different values of bright-dark scattering time *τ*_*bd*_ and fixed low temperature *T* = 10 K. With increasing intervalley scattering rate (${\tau }_{skx}^{-1}$) the degree of polarization decreases rapidly for both materials, as expected. However, the degree of valley polarization of the WSe_2_ monolayer with a dark exciton ground state is always higher than that of the MoSe_2_ monolayer, which possess a bright exciton ground state. In addition, changes in *τ*_*bd*_ do not affect the polarization degree of the exciton emission in MoSe_2_ TMDs, meaning that the kinetics is dominated by intervalley scattering at low temperature. On the other hand, the value of *τ*_*bd*_ alters efficiently the optical orientation of the darkish TMDs (WSe_2_). Fast bright-dark scattering (small value of *τ*_*bd*_) favors a high value of valley polarization, especially when the intervalley bright exciton scattering is efficient (*τ*_*skx*_ < *τ*_*rb*_ < 1). In order to understand the behavior described above, we remember that, in our model, excitons are optically created only in the bright state and then scattered either to the other valley or to a dark exciton state in the same valley. Accordingly, the valley polarization behaviors are attributed to a combination of the bright-dark exciton scattering and the intervalley relaxation. In darkish monolayers, the low-lying dark state constitutes an important reservoir which tends to maintain n_*b*_/n_*d*_ close to the expected Boltzmann distribution, in such a way that the exciton dynamics mainly take place in a single-valley rather than intervalley. Hence the total valley polarization of the exciton emission is large. In contrast, in MoSe_2_, the bright-dark exciton scattering is strongly damped, since bright excitons are the ground state. Then the intervalley scattering dominates, which depletes the population of the optically excited valley and thus reduces the degree of valley polarization.

Figure 6(b,d) show the PL intensity and the valley polarization, respectively, of the bright exciton in monolayer Mo_1−*x*_W_*x*_Se_2_ alloys as a function of temperature, for the case in which bright and dark intravalley dynamics and bright exciton intervalley scatterings are taken into account. A comparison between these results with the ones presented in Fig. 5(a), which shows the bright exciton PL intensity as a function of temperature in a model which considers only intravalley channels, unveils that the intervalley scattering slightly affects the PL intensity. In both cases, the PL intensity in bright alloys is larger than that in darkish alloys, and it is quenched in high temperature. On the other hand, a high degree of valley polarization in darkish alloys is observed, see Fig. 6(d). It decreases with a reduction of the W-concentration, indicating that the dark exciton ground state provides an important reservoir for valley polarization. This reservoir is robust because there is no exchange intervalley scattering channel for dark excitons^24,62^.

Figure 6(d) also shows that the valley polarization is strongly suppressed by increasing temperature, as the low-lying dark state in the darkish alloy, which stabilizes the valley population, becomes less relevant due to thermal intravalley excitations. We emphasize that, for a complex case in which several excitonic channels are involved in the valley dynamics, the ration between the exciton lifetime and the intervalley scattering time is not the unique factor that influences the valley polarization. However, in first order approximation, the sharp decline of the valley polarization with rising temperature can be understood as a consequence of the reduction of the scattering time *τ*_*skx*_ compared with the exciton lifetime *τ*_*rb*_, since we consider that only the latter depends on temperature. The inclusion of a temperature-dependent (phonon-mediated) intervalley scattering time should produce a smooth tail on the valley polarization for higher temperatures.

For completeness, we investigate more complicated systems corresponding to the alloys under high-intensity excitation. In this case, not only bright and dark excitons but also biexcitons involved scattering processes are taken into account, as shown in Fig. 4(d) and described by Eq. 7. Figure 6(c,e) show the PL intensity and the valley polarization of the bright exciton in monolayer Mo_1−*x*_W_*x*_Se_2_ alloys excited by a right circularly polarized continuous wave laser with a power density *P* = 1.0 kW/cm^2^, as a function of temperatures, for six different W-concentrations. It is worth recalling that Fig. 6(c,e) are similar plots to (b) and (d), but with the biexcitons being included. Note that the effect of the biexciton channel on the PL intensity of the bright exciton is the same as observed in Fig. 5(c,d), that is, the biexciton enhances significantly the stability of the exciton emission against variations of W-concentration for bright alloys. More interestingly, a comparison between plots (c) and (d) reveals that the inclusion of the biexciton channel almost does not alter the valley polarization degree, at least for alloys excited with a laser power density of *P* = 1.0 kW/cm^2^.

**Figure 7:**
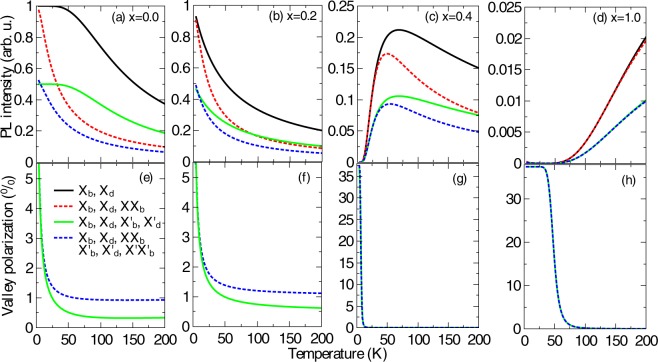
(**a**–**d**) PL intensity and (**e**–**h**) valley polarization degree of the bright exciton channel at K-valley, *X*_*b*_, of monolayer Mo_1−*x*_W_*x*_Se_2_ alloy excited with a right circularly polarized continuous wave laser as a function of temperature, for different tungsten concentrations, *x* = [0.0, 0.2, 0.4, 1.0]. We compare results obtained with the four different cases represented in Figs 3 and 4 and described in Eqs 4 to 7. The legend of panel (e) shows the excitonic channels considered in each case and applies for all the panels.

Finally, our main findings are summarized in Fig. 7, which shows the PL intensity (a–d) and the valley polarization degree (e–h) for the *X*_*b*_ channel as a function of temperature for monolayers with different compositions, MoSe_2_, Mo_0.6_W_0.4_Se_2_, Mo_0.4_W_0.6_Se_2_, and WSe_2_. In order to make a more complete comparison between the four different cases shown in Fig. 4, for a certain W-concentration, we illustrate an evolution of the curves via progressively adding more and more scattering channels. It is worthy to remind that all the PL intensities are normalized by the same factor so that it is possible to contrast the relative intensity between different cases. By comparing distinct W-concentrations, we observe the three different regimes of the PL intensity as a function of temperature. It decreases with rising *T* for bright alloys [panels (a) and (b)] and increases with *T* in darkish alloys [panel (d)], while shows a non-monotonic behavior for intermediate W-concentrations [panel (c)]. The same trend of the PL intensity with rising temperature is observed for a given *x* irrespective of the channels involved in the valley dynamics. Furthermore, the observation of the different panels unveils that the overall PL intensity decreases with an increase of *x* [note the different scales in panels (a) to (d)].

Figure 7(a–d) also shows that, for a fixed composition, the PL intensity related to the radiative recombination of bright excitons in a given valley decreases when we take into account intervalley scattering channels, as expected. In addition, the inclusion, in our model, of the Auger-type exciton-exciton annihilation process that creates biexcitons leads to a faster (slower) decrease (increase) of the PL intensity as the temperature rises for bright (darkish) alloys. The valley polarization, on the other hand, is less sensitive to the inclusion of biexciton channels for an alloy with any W-concentration, as shown in panels (e) to (h).

## Conclusion

The spin splitting around either K- or K′ in the conduction band of ternary Mo_1−*x*_W_*x*_Se_2_ alloy monolayers can be tuned in a wide range - its sign is even reversed from positive to negative values as tungsten-concentration (*x*) increases from zero to one. Since the sign of spin splitting determines the nature of the exciton ground state as optically active (bright) or passive (dark), the character of the ground states can be manipulated by the value of *x*. We propose a comprehensive theory based on a set of rate equations to study the valley dynamics in ternary alloy monolayers as the alloy varies continuously from MoSe_2_ to WSe_2_. To compare with experimental data, in a steady state, we have calculated two experimentally observable quantities, the PL intensity and the valley polarization, in a wide range of temperatures. By systematically adding more and more scattering or recombination channels, we quantitatively determine the contribution of each scattering channel such as dark exciton, biexciton, intervalley scattering between bright excitons, and bright biexcitons to the valley dynamics. We find that the PL intensity of an exciton emission decreases by cooling a WX_2_ monolayer, nevertheless, an opposite behavior is shown in MoX_2_ compounds, which is in good agreement with experimental observations. We demonstrate that the alloys can be classified into three groups according to the dependence of the excitonic PL intensity on temperature: MoSe_2_-like alloys in which the PL intensity decreases with increasing temperature, WSe_2_-like alloys where the PL intensity increases with rising temperature, and MoSe_2_ and WSe_2_ mixed alloys in which a transition from the MoSe_2_-like to WSe_2_-like takes place, showing a non-monotonic temperature dependence.

Because the low-lying dark state in darkish alloys quenches the PL intensity at low temperatures, it stabilizes the intravalley population, enhancing the valley polarization. Bright alloys, on the other hand, show high PL intensity, but low valley polarization. Our results reveal that, by properly selecting the value of *x*, an alloy with considerably strong PL intensity and a high valley polarization is achievable. For an alloy with *x* = 0.4, for instance, the PL intensity of the bright exciton is around three times larger than the corresponding PL intensity of WSe_2_ binary monolayers (*x* = 1) at room temperature. Simultaneously, the valley polarization of this alloy is twice larger than the VP of MoSe_2_ monolayers (*x* = 0). This scenario sheds light on high-performance valleytronic, photonic and optoelectronic devices, which demands simultaneously an efficient optical emission and a high valley polarization. Finally, we also notice that biexciton enhances the stability of the exciton emission against a fluctuation of W-concentration for bright alloys, specially at high temperatures.

## Supplementary information

Supplementary Material for “Dark-exciton valley dynamics in transition metal dichalcogenide alloy monolayers”
