# Supplementary material for: Dark-exciton valley dynamics in transition metal dichalcogenide alloy monolayers
Source: Sci Rep. 2019 Mar 14;9:4575. doi: 10.1038/s41598-019-40932-9 (PMC6418264; doi:10.1038/s41598-019-40932-9)
Supplement: Supplementary file 1 — Supplementary Material for “Dark-exciton valley dynamics in transition metal dichalcogenide alloy monolayers” [file 41598_2019_40932_MOESM1_ESM.pdf]

Supplementary Material for "Dark-exciton valley dynamics in transition metal dichalcogenide alloy monolayers"

Helena Bragança,[1,2] Flávio Riche,[2] Fanyao Qu,[2] Victor Lopez-Richard,[1] Gilmar Eugenio Marques[1]

[1]*Departamento de Física, Universidade Federal de São Carlos, Brazil*
[2]*Instituto de Física, Universidade de Brasília, Brasília-DF 70919-970, Brazil*

I. INFLUENCE OF INDIRECT EXCITONS ON TMD MONOLAYERS

The complex electronic band structure of TMD monolayers comprising several spin-split valleys in the conduction and valence bands leads to the formation of dark excitonic states. Although inaccessible by light due to the required spin-flip or momentum transfer, they have an important impact on the excitonic optical response of the monolayers. In the main text, we have analyzed the exciton dynamics in ternary monolayer $Mo_{1-x}W_xSe_2$ alloys involving spin-forbidden intravalley dark excitons. For completeness, here we also include indirect dark-exciton, with non-zero center-of-mass momentum, such as $X_{KK'}$ ($X_{K'K}$) which consists of the hole located in the K (K') and the electron in the K' (K) valley, as shown in Fig. 1(a) and (b). Similar to the spin-forbidden dark excitons, the energy of the indirect exciton can be either higher or lower than that of the bright exciton depending on the tungsten concentration [see Fig. 1(a) and (b)].

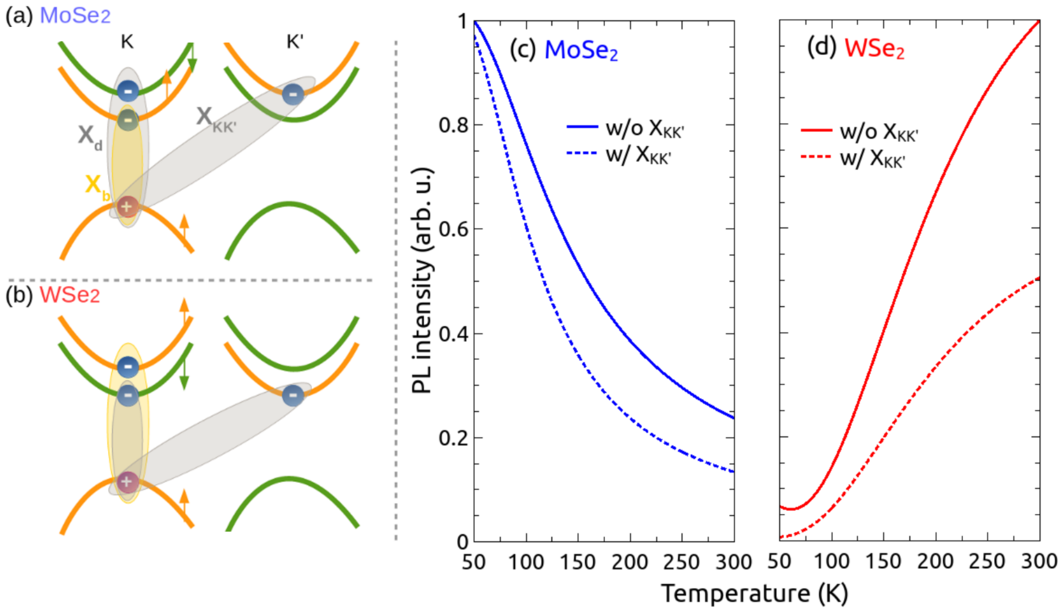

FIG. 1. Bright (X_b), spin-forbidden (X_d) and momentum forbidden ($X_{KK'}$) excitons in TMD monolayers. (a)-(b) Schematic representation of single-particle dispersion for $MoSe_2$ and WSe_2 monolayers, showing the electron-hole quasiparticles for the considered excitonic transitions around the K, K$'$ valleys. Orange and green bands represent states with spin up and spin down, respectively. (c)-(d) PL intensity of intravalley bright excitons (X_b) in the K valley as a function of temperature for $MoSe_2$ and WSe_2 monolayers. Solid lines correspond to results obtained with just one kind of dark exciton channel, the intravalley spin-forbidden one. Dotted lines, on the other hand, were obtained in the presence of both spin-forbidden and momentum-forbidden dark excitons.

In order to clearly present the dynamics of bright and dark states including both spin- and momentum-forbidden excitons we extend the set of rate equations described in Eq. (6) of the main text for bright and intravalley dark excitons in both K and K' valleys by including new excitonic channels related to scatterings between bright and indirect excitons. The complete valley dynamics is governed by the following set of rate equations

$$\frac{dn_b}{dt} = g - \frac{n_b}{\tau_{rb}} - \frac{n_b}{\tau_{skx}} + \frac{n_{b'}}{\tau_{skx}} - \frac{n_b}{\tau_{bd}}\Theta_{(x_c-x)} + \frac{n_d}{\tau_{bd}}\Theta_{(x-x_c)} - \frac{n_b}{\tau_v}\Theta_{(x_c-x)} + \frac{n_{KK'}}{\tau_v}\Theta_{(x-x_c)}$$
$$\frac{dn_{b'}}{dt} = g' - \frac{n_{b'}}{\tau_{rb}} - \frac{n_{b'}}{\tau_{skx}} + \frac{n_b}{\tau_{skx}} - \frac{n_{b'}}{\tau_{bd}}\Theta_{(x_c-x)} + \frac{n_{d'}}{\tau_{bd}}\Theta_{(x-x_c)} - \frac{n_b'}{\tau_v}\Theta_{(x_c-x)} + \frac{n_{K'K}}{\tau_v}\Theta_{(x-x_c)}$$
$$\frac{dn_d}{dt} = -\frac{n_d}{\tau_{rd}} - \frac{n_d}{\tau_{bd}}\Theta_{(x-x_c)} + \frac{n_b}{\tau_{bd}}\Theta_{(x_c-x)}$$
$$\frac{dn_{d'}}{dt} = -\frac{n_{d'}}{\tau_{rd}} - \frac{n_{d'}}{\tau_{bd}}\Theta_{(x-x_c)} + \frac{n_{b'}}{\tau_{bd}}\Theta_{(x_c-x)}$$
$$\frac{dn_{KK'}}{dt} = -\frac{n_{KK'}}{\tau_{rd}} - \frac{n_{KK'}}{\tau_v}\Theta_{(x-x_c)} + \frac{n_b}{\tau_v}\Theta_{(x_c-x)}$$
$$\frac{dn_{K'K}}{dt} = -\frac{n_{K'K}}{\tau_{rd}} - \frac{n_{K'K}}{\tau_v}\Theta_{(x-x_c)} + \frac{n_b'}{\tau_v}\Theta_{(x_c-x)}. \quad (1)$$

As described in the main text, the rate equations include photon-generation of intravalley bright excitons (g), the radiative (τ_{rb}) and non-radiative (τ_{rd}) recombinations of bright and dark excitons to the ground-state, bright-dark intravalley (τ_{bd}) and intervalley (τ_{skx}) scatterings between bright excitons. We also include scatterings between the direct bright exciton X_b (X_b') and the indirect $X_{KK'}$ ($X_{K'K}$) one, with concentration $n_{\mathrm{K,K'}}$ ($n_{\mathrm{K',K}}$) and lifetime τ_v. This process involves phonon-mediated scatterings of electrons in the conduction band and does not require a spin flip, thus is more efficient than the spin-flip bright-dark intravalley scattering process. While the latter occurs in a picosecond timescale, the former happens in a sub-picosecond scale, that is, $\tau_v = 0.1$ ps [1], the other relevant parameters are displayed in Table I of the main text.

Figure 1(c) and (d) show the PL intensities of the bright excitons in K valley for both $MoSe_2$ and WSe_2 monolayers respectively, as a function of temperature. Interestingly, a comparison between results obtained without (solid line) and with (dotted line) indirect excitons shows the same trend of the PL intensity dependence on temperature, that is, it decreases with rising temperature in $MoSe_2$ monolayers and increases for WSe_2. The opposite behavior for Mo- and W-based material can be explained by the different alignment of bright and dark excitons, which determines the thermal equilibrium population of each excitonic state, as explained in the main text. The main effect of indirect excitons is only a small reduction of the PL intensity of bright excitons, due to the presence of a new channel $X_{KK'}$ ($X_{K'K}$) with long lifetime (τ_{rd}, due to the non-radiative recombination) that holds part of the exciton population.

II. BIEXCITON FINE STRUCTURE

As described in the main text, besides single excitons, more complex excitonic states can be formed in TMD binary and ternary monolayers. Due to strong Coulomb interactions, two excitons can bind together to form a four-particle complex, the biexciton, analogous to the H_2 molecule. Recently, extensive experimental studies about the biexciton formation have been realized. It has been reported that, apart from the usual biexciton composed of two bright excitons, the biexcitons comprised of one bright and one dark exciton can also be formed in TMD monolayers [2]. A schematic representation of the intravalley biexciton fine structure in the K valley is shown in Fig. 2(a) and (b), for $MoSe_2$ and WSe_2, respectively.

The exciton dynamics including intravalley bright-bright biexcitons were analyzed in the main text, throughout Eq. (7) presented therein. Here we extend the model to include bright-dark intravalley biexcitons:

$$
\begin{aligned}
\frac{dn_b}{dt} &= g - \frac{n_b}{\tau_{rb}} - \frac{n_b}{\tau_{skx}} + \frac{n_{b'}}{\tau_{skx}} - \frac{n_b}{\tau_{bd}}\Theta_{(x_c - x)} \\
&\quad + \frac{n_d}{\tau_{bd}}\Theta_{(x-x_c)} + \frac{n_{bb}}{\tau_{rbb}} - 2\beta n_b^2 - \beta n_b n_d \\
\frac{dn_{b'}}{dt} &= g' - \frac{n_{b'}}{\tau_{rb}} - \frac{n_{b'}}{\tau_{skx}} + \frac{n_b}{\tau_{skx}} - \frac{n_{b'}}{\tau_{bd}}\Theta_{(x_c - x)} \\
&\quad + \frac{n_{d'}}{\tau_{bd}}\Theta_{(x-x_c)} + \frac{n_{b'b'}}{\tau_{rbb}} - 2\beta n_{b'}^2 - \beta n_b' n_d' \\
\frac{dn_d}{dt} &= -\frac{n_d}{\tau_{rd}} - \frac{n_d}{\tau_{bd}}\Theta_{(x-x_c)} + \frac{n_b}{\tau_{bd}}\Theta_{(x_c-x)} + \frac{n_{bd}}{\tau_{rbb}} - \beta n_b n_d \\
\frac{dn_{d'}}{dt} &= -\frac{n_{d'}}{\tau_{rd}} - \frac{n_{d'}}{\tau_{bd}}\Theta_{(x-x_c)} + \frac{n_{b'}}{\tau_{bd}}\Theta_{(x_c-x)} + \frac{n_{b'd'}}{\tau_{rbb}} - \beta n_b' n_d' \\
\frac{dn_{bb}}{dt} &= -\frac{n_{bb}}{\tau_{skxx}} - \frac{n_{bb}}{\tau_{rbb}} + \frac{n_{b'b'}}{\tau_{skxx}} + \beta n_b^2 \\
\frac{dn_{b'b'}}{dt} &= -\frac{n_{b'b'}}{\tau_{skxx}} - \frac{n_{b'b'}}{\tau_{rbb}} + \frac{n_{bb}}{\tau_{skxx}} + \beta n_{b'}^2 \\
\frac{dn_{bd}}{dt} &= -\frac{n_{bd}}{\tau_{rbb}} + \beta n_b n_d \\
\frac{dn_{b'd'}}{dt} &= -\frac{n_{b'd'}}{\tau_{rbb}} + \beta n_b' n_d'
\end{aligned} \qquad (2)
$$

Besides the scattering and recombination channels described in the main text, we add the bright-dark biexcitons, whose density is represented by n_{bd} (the ' index stand for quasi-particles in the K' valley). After its formation, the bright-dark biexciton radiatively recombines through its bright component (the final state is a dark exciton), then the recombination time (τ_{rbb}) is equal for both biexcitons.

Interestingly, our results indicate that the PL intensity of intervalley bright excitons is essentially insensitive to the inclusion of the extra bright-dark biexciton channel in the exciton dynamics. Fig. 2(c) shows the PL intensity of intravalley bright excitons in the K valley as a function of temperature for different TMD monolayers. The PL intensities obtained in the presence of the bright-dark biexciton channel are only slightly smaller than the ones obtained in the absence of this channel.

---

[1] Malte Selig, Gunnar Berghauser, Marten Richter, Rudolf Bratschitsch, Andreas Knorr, and Ermin Malic, "Dark and bright exciton formation, thermalization, and photoluminescence in monolayer transition metal dichalcogenides", 2D Materials **5**, 3 (2018).
[2] Cedric Robert, "When bright and dark bind together", Nature Nanotechnology **13**, 982983 (2018).

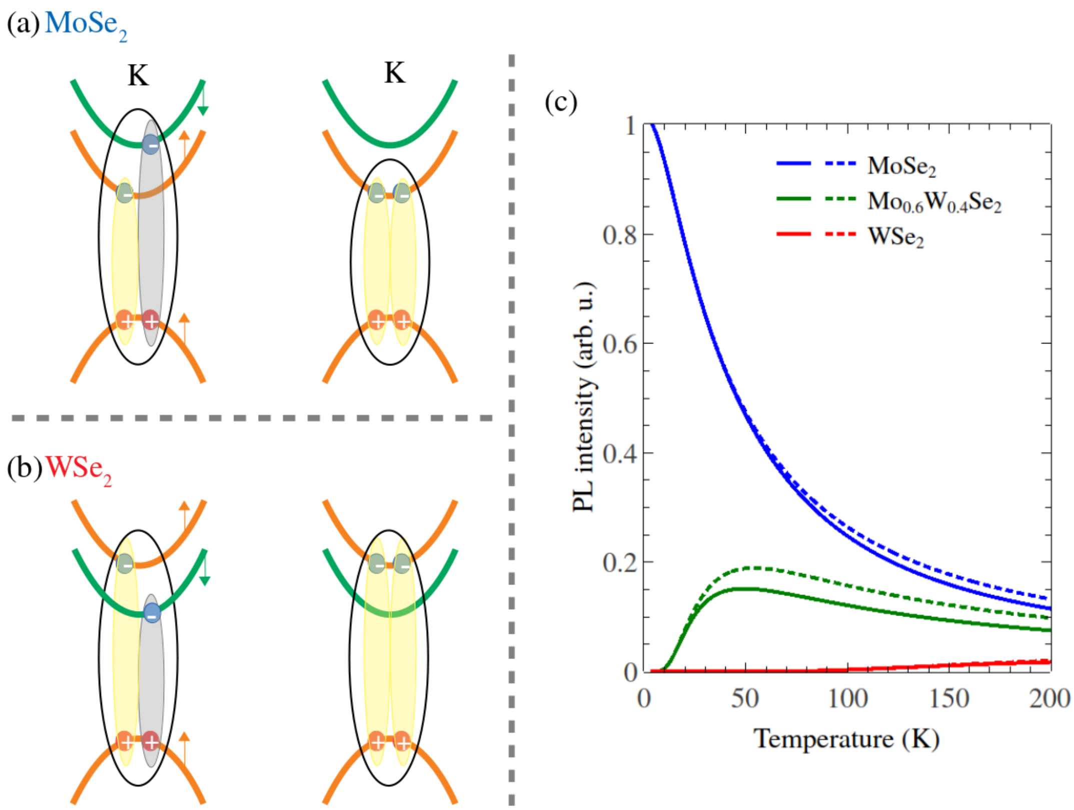

FIG. 2. Fine structure of intravalley biexcitons in (a) $MoSe_2$ and (b) WSe_2 monolayers. We show (left-hand side) bright-dark intravalley and (right-hand side) bright-bright intravalley biexcitons, both in the K valley. (c) PL intensity of intravalley bright excitons in the K valley as a function of temperature for different TMD monolayers. Solid lines correspond to results obtained in the presence of both bright-dark and bright-bright biexcitons, while dotted lines were obtained in the presence of only bright-bright biexcitons.
